# Retracted: Epidemiological Pattern of Newly Diagnosed Children with Type 1 Diabetes Mellitus, Taif, Saudi Arabia

**DOI:** 10.1155/2016/6736029

**Published:** 2016-08-18

**Authors:** The Scientific World Journal

At the request of the authors and the institution, the article titled “Epidemiological Pattern of Newly Diagnosed Children with Type 1 Diabetes Mellitus, Taif, Saudi Arabia” [1] has been retracted. The article was found to contain a substantial amount of material, without citation, from an M.D. thesis titled “A study of the etiology, risk factors, clinical features and pitfalls in management of newly diagnosed diabetic children and adolescents,” by Abeer Mohamed ElSayed Osman.
